# Le lupus systémique juvénile familial: à propos de deux familles

**DOI:** 10.11604/pamj.2015.20.419.5735

**Published:** 2015-04-29

**Authors:** Sanaa Krich, Kawtar Inani, Mariame Meziane, Fatima Zohta Souilmi, Samir Atmani, Mustapha Hida, Taoufik Harmouch, Afaf Amarti, Fatima Zohra Mernissi

**Affiliations:** 1 Service de Dermatologie, CHU Hassan II, Fès, Maroc; 2 Service de Pédiatrie, CHU Hassan II, Fès, Maroc; 3 Service d'Anatomopathologie, CHU Hassan II, Fès, Maroc

**Keywords:** Lupus érythémateux systémique, juvénile, familial, Maroc, LLupus erythemateux systemique, juvenile, family, Morocco

## Abstract

Le lupus érythémateux systémique (LES) juvénile est une connectivite rare, d’évolution plus sévère que chez l'adulte. Les cas familiaux sont exceptionnels. Il s'agissait de deux familles (5 patients atteints), chez qui on a objectivé un LES juvénile chez deux sæurs âgées de 14 ans et 6 ans respectivement chez la première famille, deux frères, âgés de 20 ans et 6 ans respectivement plus une sæur âgée de 10 ans chez la deuxième famille. Dans tout les cas le diagnostic de lupus systémique a été posé selon les nouveaux critères du groupe du SLICC (Systemic Lupus International Collaborating Clinics). Tous les patients ont été mis sous photoprotection, dermocorticoïdes et antipaludéens de synthèse sauf dans deux cas où la corticothérapie a été administrée. L’évolution a été favorable après le traitement dans la majorité des cas. Nous rapportons ces cas afin de discuter les particularités épidémiologiques, physiopathologiques, cliniques, paracliniques et thérapeutiques.

## Introduction

Le LES juvénile est une connectivite rare, d’évolution plus sévère que chez l'adulte [1]. Il est souvent sporadique et les cas familiaux sont exceptionnels (10-15%) [2]. Peu d’études de lupus familial particulièrement juvénile ont été rapporté d'où l'intérêt de ce travail pour discuter les particularités épidémiologique, physiopathologique, cliniques, paracliniques et thérapeutiques par rapport aux données de la littérature.

## Patient et observation

### Famille 1

**Figure 1:**
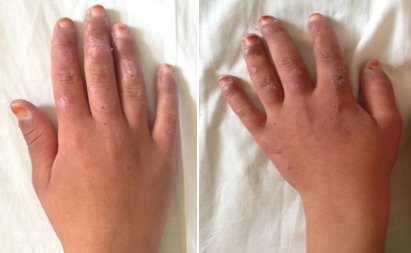
Plaques érythémato-squameuses de lupus discoide au niveau des espaces interarticulaires

**Figure 2:**
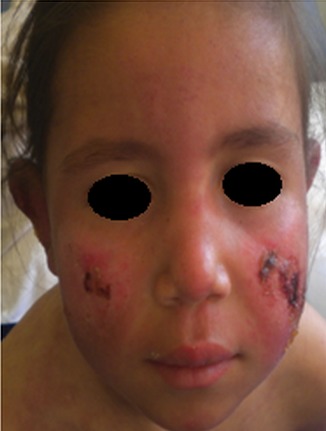
Placards érythémato-croûteux malaires

**Figure 3:**
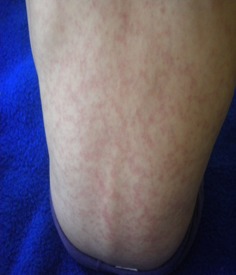
Un livido réticulé du dos

**Figure 4:**
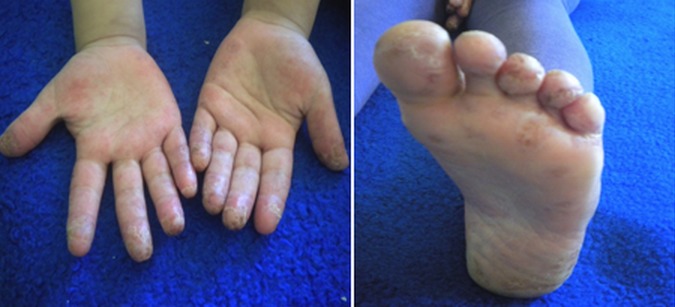
Lésions de pulpites des mains et pieds

Il s'agissait de deux sœurs issues d'un mariage consanguin de 1er degré. L'ainée était âgée de 14 ans (sœur 1.1), 3^ème^ d′une fratrie de 4 et présentait depuis l’âge de 6ans une photosensibilité, des arthralgies inflammatoires, un rash malaire avec une pigmentation séquellaire, des lésions de lupus discoïde des espaces interarticulaires (EIA) des mains ainsi qu'une pulpite des mains et des pieds (Figure 1). La benjamine était âgée de 7ans (sœur 1.2) dont le début de la symptomatologie remontait à l’âge de 5 ans et demi par une photosensibilité, des arthralgies inflammatoires, des érosions buccales et rash malaire surmonté d’érosions croûteuses par endroit (Figure 2) et au niveau des oreilles d’évolutions estivales avec un livédo réticulé non infiltré au niveau des membres et le tronc (Figure 3) et des pulpites des mains et pieds (Figure 4).

### Famille 2

**Figure 5:**
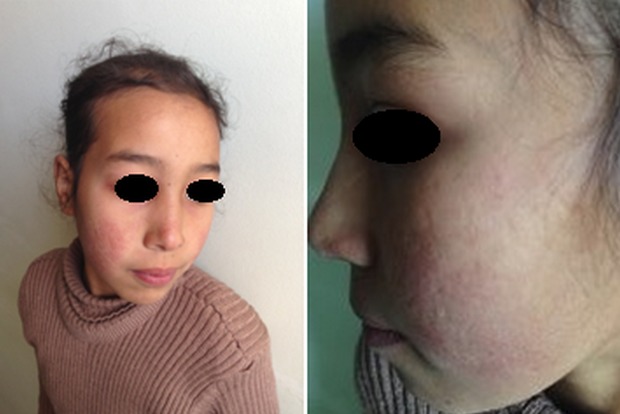
Rash malaire avec des cicatrices atrophiques

Il s'agissait de deux frères et une sœur issue d'un mariage consanguin de 3ème degré dont l'ainé d'une fratrie de 4 (frère 2.1), était âgé de 20 ans et qui présentait depuis l’âge de 16 ans une photosensibilité, des arthralgies inflammatoires, un rash malaire, des pulpites des mains et pieds ainsi que des érosions au niveau des oreilles d'aggravation hivernale. Son frère benjamin (frère 2.2) âgé de 6 ans avait une notion d'otites à répétition ainsi qu'une photosensibilité et arthralgies inflammatoires depuis 7 mois. L'examen clinique retrouvait des lésions de lupus discoïde au niveau du visage et des EIA des mains ainsi que des pulpites des mains et des pieds. Concernant la sœur (sœur 2.3), qui était la 3ème de la fratrie, elle était âgée de 10 ans et qui présentait depuis l’âge de 6 ans, une photosensibilité, un rash malaire avec des cicatrices atrophiques (Figure 5) et des pulpites des mains et des pieds. Les données, cliniques, biologiques, radiologiques, immunologiques, histologiques, thérapeutique ainsi que l’évolution sont résumés ci-dessous dans le Tableau 1.

**Tableau 1:** Les données, cliniques, biologiques, radiologiques, immunologiques, histologiques, thérapeutique ainsi que l’évolution des cas dans les deux familles

Données	cliniques	biologiques	radiologiques	Immunologiques	histologiques	thérapeutiques	évolution
**Famille N° 1**							
-sœur 1.1	Macules pigmentées (visage) Pulpites Lésions de lupus discoïde en EIA des mains	Syndrome inflammatoire C4 diminué Hématurie Prot 24h négative	Radiographie Thorax: N ETT: N Test de couleur + CV:N	ANN, APL et anti-SSa: positifs	BC: lupus discoïde PBR: lupus Rénal stade I (OMS)	Dermocorticoïdes Hydroxychloroquine 6.5 mg/kg/j Aspégic 100mg/j	Favorable après un recul de 6mois puis PDV
-sœur 1.2	Rash malaire Erosions croûteuses (visage + oreille) Pulpites Livido(dos + MI) Erosions buccales	Syndrome Inflammatoire Anémie Lymphopénie C4 diminué Protéinurie de 24h positive	Radiographie Thorax: N ETT: N Test de couleur + CV:N	ANN, anti-DNA et anti-SSa: positifs	BC: lupus Subaigu PBR: lupus Rénal stade II (OMS)	Dermocorticoïdes Hydroxyhchloroquine 6.5 mg/kg/j Bolus de corticoïdes (3js) puis relais/ Prednisone 2mg/kg/j	Favorable après un recul de 1 an actuellement sous 7,5 mg/j de Prednisone
**Famille N°2**							
-frère 2.1	Rash malaire Pulpites des extrémités Erosion au niveau des oreilles	Syndrome inflammatoire C3 diminué Prot 24 h négative	Radiographie Thorax: N Test de couleur + CV:N ECG: N	AAN positifs	BC: IFD positive en faveur d'un lupus	Hydroxychloroquine 6,5mg/kg/j	En cours
-frère 2.2	Lésions de lupus discoïde en ailes de papillon au niveau du visage et en EIA des mains + pulpites	Syndrome Inflammatoire Anémie, Prot 24h Négative	Test de Couleur + CV:N ECG: N	AAN et anti-SSa: Positifs		Aspégic Dermocorticoïdes Puis Hydroxychloroquine 6.5 mg/kg/j	Amélioration des arthralgies et aggravations des lésions cutanées après 1 an d'où la mise sous prednisone 0,5mg/kg/j
-sœur 2.3	Rash malaire Erythème eczématiforme des EIA des mains Pulpites	Syndrome Inflammatoire Prot 24h Négative	Radiographie Thorax: N Test de couleur + CV:N ECG: N	AAN et anti-DNA: Positifs	BC: Sensiblement Normale	Hydroxychloroquine 6,5mg/kg/j	En cours

AAN: anticorps anti-nucléaires; APL: anticorps anti-phospholipides; C4: complément 4; C3: complément 3; N: normal; CV: champs visuel; ETT: échographie cardiaque; BC: biopsie cutanée; PBR: ponction biopsie rénale; OMS: organisation mondiale de la santé; EIA: espace inter-articulaire; MI: membres inférieurs; ECG: électrocardiogramme. IFD: immunofluorescence directe; Prot 24h: protéinurie de 24 h. N: normal.

## Discussion

Nous rapportons des premières observations de LES juvénile familial intéressant des familles marocaines avec notion de consanguinité. A notre connaissance aucun cas n'a été rapporté au nord d'Afrique, cependant des études rétrospective de LES juvénile ont été rapporté dans des pays orientaux comme l'Egypte, l'Arabie Saoudite, Kuwait, Oman et Bahreïn où les cas familiaux ont été fréquemment rapporté par rapport à la population caucasienne [3].

Le LES est une maladie auto-immune non spécifique d'organe résultant de l'interaction de plusieurs facteurs génétiques et environnementaux. Il est caractérisé par la production d'une vaste gamme d'auto-anticorps principalement dirigés contre les antigènes nucléaires et les complexes immuns, qui peuvent conduire à l'atteinte de plusieurs organes. Le LES débute à l’âge pédiatrique dans environ 20% des cas [4, 5].

Ce lupus juvénile est une pathologie rare, même s'il est considéré parmi les connectivites fréquentes de l'enfant [6]. Son diagnostic est porté avant l’âge de 16 ans dans 20% des cas et peut également toucher le jeune enfant chez qui un contexte familial peut être associé comme nos cas. Le sex-ratio fille/garçon semble moins élevé que chez l'adulte et varie considérablement d'une étude à l'autre de 1/5 à 1/18 [7]; il est plus faible avant la puberté qu'après. Nos résultats sont discordants de la littérature puisque le sex-ratio fille/garçon était plus élevé à 3/2. La fréquence de la maladie varie également selon les ethnies [8] et la géographie [9].

Sur le plan clinique les manifestations initiales sont polymorphes et parfois trompeuses. Au début de la maladie, un seul organe peut être atteint, mais la forme systémique est la forme de révélation habituelle. Les signes cutanéo-muqueux peuvent être spécifiques ou non. Les lésions bulleuses, le lupus discoïde, l'alopécie et le phénomène de Raynaud sont rares [6]. Dans nos cas le rash malaire, la photosensibilité et les pulpites étaient les manifestations cutanées les plus fréquentes. Deux cas de lupus discoïde ont été notés. Dans la littérature, l'atteinte des autres organes au cours du LES juvénile est dominée par l'atteinte rénale (30 à 80%) qui peut être sévère d'emblée. Ceci a été objectivé chez une de nos patientes (sœur 1.2) qui a présenté une atteinte rénale stade II selon la classification de l'OMS nécessitant des bolus de corticothérapie puis relais par corticothérapie 2mg/kg/j. les autres atteintes rapportées dans les études après l'atteinte rénale sont: l'atteinte articulaire dans 80%, l'atteinte neuropsychiatrique (20 - 95%) et l'atteinte cardio-pulmonaire (5 – 30%) [6]. Ces données concordent avec nos cas où tous les cas ont présenté une atteinte articulaire.

Les anomalies biologiques les plus fréquentes sont [7]: la protéinurie, l'hématurie, les troubles hématologiques (anémie, thrombopénie et lymphopénie) et l'hypocomplémentémie. Certains anticorps sont plus fréquents dans le LES juvénile en plus des anti-DNA, en particulier les anticorps anti-Sm, anti-RNP et anti-phospholipides (APL). Les anticorps anti- SSa peuvent également être positifs dans 14 à 40% [7]. La positivité des APL était fréquemment rapportés dans la littérature, cependant les manifestations thrombotiques étaient rares comme dans notre cas (sœur 1.1) qui avait des APL positifs sans notion de thrombose. Nos résultats immuno-biologiques concordent avec la littérature où on a constaté une fréquence des atteintes hématologiques (2/5), d'hypocomplémentémie (3/5), AAN (tous les cas), anti-DNA positifs (2/5 cas), anti-SSa (3 cas) et APL (1cas).

La prise en charge thérapeutique est multidisciplinaire [10]. En fait il n'y a pas de recommandations thérapeutiques codifiées, cependant les doses des traitements systémiques sont généralement plus élevées que chez les adultes en raison de la grande sévérité des atteintes. Grâce aux traitements actuels, l’évolution est généralement favorable. La survie après 10 ans d’évolution est environ de 90% dans les séries les plus récentes. La cause principale de mortalité est liée aux complications infectieuses, à l'insuffisance rénale chronique, à la maladie elle même, mais surtout aux effets secondaires des traitements [11].

Bien que la pathogénie de la maladie reste mal comprise, la prédisposition génétique est probablement le plus grand facteur de risque de LES. Ceci a été prouvé par les taux élevés de LES observés chez les jumeaux homozygotes [12], par l'incidence relativement élevée des cas familiaux (10-15%) [2] et par une incidence plus grande chez les Africains noirs et les Asiatiques que chez les blancs caucasiens [8]. Pour notre part nous rapportons des observations de LES juvénile avec un caractère familial et une notion de consanguinité. Ceci était également un facteur de risque remarquable dans plusieurs études [3].

Nombreuses études de génome ont mis en évidence plusieurs marqueurs phénotypiques associés à un risque élevé de LES ainsi que d'autres pathologies auto-immunes [13]. En effet, cette hétérogénéité phénotypique pourrait correspondre à une hétérogénéité génétique. Certaines formes sont polygénique, alors que d'autres sont monogéniques. Des hypothèses suggèrent que le lupus juvénile est plutôt monogénique que chez l'adulte [14]. L’étude des formes précoces, familiales ont permis la description de nouvelles causes monogéniques de lupus comme les déficits du complément, les déficits de l'apoptose et les interféronopathies [14]. Dans nos cas nous avons pu objectiver que le déficit en complément qui est fortement associé à la survenue des manifestations lupiques systémiques surtout rénales (30%) et qui peut atteindre 93% des cas pour le déficit en C1q, 60 à 66% pour le déficit en C1r/s et 75% pour le C4 [15]. Ce déficit en complément pourrait être associé à des infections à répétition [16]. Ce qui a été constaté chez un seul cas qui a présenté des otites à répétition.

## Conclusion

Le LES juvénile est une connectivite rare, d’évolution plus sévère que chez l'adulte. Les formes de début sont trompeuses d'où l'intérêt de rechercher des formes familiales devant tous les cas de lupus juvénile afin de démarrer une prise en charge précoce pour éviter les complications.
